# Strengthening clinical development activities and preparedness for vaccine manufacturers from emerging countries: Results of a survey

**DOI:** 10.1016/j.jvacx.2022.100255

**Published:** 2022-12-28

**Authors:** Simonetta Viviani, Paul Willems, Sonia Pagliusi

**Affiliations:** a Vaccines, Epidemiology & Public Health, Via Gramsci 12, Loc.Strove, 53035 Monteriggioni, Siena, Italy; b Independent Expert in Clinical Development, Safety & Pharmacovigilance; c DCVMN International, Route de Crassier 7, 1262 Eysinns-Nyon, Switzerland

**Keywords:** Clinical studies, Epidemiology, Ethics, Safety monitoring, Preparedness, Epidemic, Pandemic

## Abstract

Vaccine development, in most cases, is a long, complex process, often lasting years and involving a combination of public and private stakeholders. Particularly, the vaccine clinical development process is highly regulated by several guidelines, regulatory pathways and science-based recommendations from experts. Designing and executing a successful clinical development plan for any candidate vaccine requires a solid scientific, medical, operational and regulatory knowledge and expertise, to comply with regulations and assure adequate benefit-risk balance for the product to be used in mass vaccination of healthy populations.

The purpose of this study was to assess the approaches and practices related to Clinical Development functions, and related activities among vaccine manufacturers based in emerging countries, and to identify industry needs in terms of organizational development and training needs. A structured questionnaire designed specifically for assessing indicators of clinical activities, in the last five years, comprised of four sections aimed to collect information on (1) the organizational structure and the activities conducted by the clinical functions; (2) the clinical trial design ability and the management of clinical trial documents; (3) the clinical trial management and monitoring activities; (4) the quality aspects of clinical activities. The results suggest that the great majority of respondents is engaged in intense clinical development activities, as indicated by the high number of licensed vaccines available and supplied in the national markets or in foreign markets, including vaccines with WHO prequalification status.

Areas to further strengthen the clinical activities and medical research preparedness were identified. Greater engagements of stakeholders’ and investments will be required to expand the clinical basis in vaccine R&D, and to support achieving a high level of preparedness in emerging countries, for development of new vaccines against future regional epidemics and global pandemics.

## Introduction

Safe and effective vaccines are important and cost-effective tools to control the impact of infectious diseases, in endemic, epidemic and pandemic settings, aimed for local, regional and/or global use [1], by preventing morbidity and mortality related to diseases. Vaccine development, in most cases, is a long, complex process, often lasting years and involving a combination of public and private stakeholders [2]. The clinical product development plan [3] is an essential component of vaccine development to obtain licensure and marketing authorization (MA) of new vaccines. Vaccine clinical development is highly regulated by several guidelines, regulatory pathways and science-based recommendations from experts. Designing and executing a successful clinical development plan for any candidate vaccine requires a solid scientific, medical, operational and regulatory knowledge and expertise, to comply with regulations [4] and assure adequate benefit-risk balance for the product to be used in mass vaccination of healthy populations. However, not all stakeholders may be able to skillfully navigate through the clinical development and assessment of vaccine candidates and the facilitated pathways offered by the various regulatory agencies [5].

In general, the field of healthcare, medical research and clinical trials take place within an established framework of professional ethical standards. The worldwide reference of ethical guidelines is the Declaration of Helsinki on Ethical Principles for Medical Research Involving Human Subjects, issued by the World Medical Association and periodically revised since the first edition published in 1964 [6]. Other recognized ethical guidelines are issued by the International Conference for Harmonization (ICH) [7] the World Health Organization (WHO) [8] and by other National Regulatory Authorities (NRAs). Other important guidelines covering general scientific and methodological aspects of clinical development and research, applicable to vaccine clinical development, are those issued by ICH with E6 (Good Clinical Practices), E8 (General Considerations for Clinical Trials), E9 (Statistical Principles for Clinical Trials), E3 (Clinical Study Reports) and others under the ICH efficacy-guidelines framework [9]. In addition, comprehensive references, guidelines and recommendations concerning several aspects of vaccine development including vaccine specific clinical development are issued by WHO [10], European Medicinal Agency (EMA) [11], US Food Drug Administration (FDA) [12] and other NRAs, as well as the several working group consensus guidance documents of the Council for International Organizations of Medical Sciences (CIOMS).^1^

Furthermore, preparedness to fight pandemic and epidemic threats also relates to the preparedness in planning, implementing and interpreting clinical studies to produce, supply and deliver safe and effective vaccines.

The Developing Countries' Vaccine Manufacturers Network (DCVMN) is a voluntary public health-driven alliance of vaccine manufacturers based in 14 developing countries, engaged in research, development, manufacturing and supply of high-quality vaccines to protect people against known and emerging infectious diseases globally.^2^ The DCVMN secretariat was tasked by its members with the strategic goal of seeking solutions, jointly with manufacturers, for enabling the stable, sustainable supply of quality vaccines to prevent infectious diseases in developing countries. As vaccines are given to millions of healthy people, including children, to prevent life-threatening diseases, vaccines must meet high safety and efficacy levels required by regulatory authorities. The purpose of this study was to assess the approaches and practices related to clinical development functions, activities and regulatory expertise available among vaccine manufacturers based in emerging countries, to identify industry needs, particularly among the vaccine manufacturers members of the DCVMN network. The survey questions, were designed to assess indicators of clinical development activities, with some overlap to regulatory activities, to guide future training support and best practice sharing for vaccine manufacturers.

## Methods

The study was based on a structured questionnaire designed by a senior clinical expert specifically for assessing indicators of clinical activities. The survey was circulated to members of the Developing Countries Vaccine Manufacturers Network (DCVMN) and was completed between 1st July and 15th August 2021 through invitations by email, with weekly reminders, to all 41 DCVMN members at the time of the survey, sharing the structured questionnaire designed specifically for this purpose. The invitation to manufacturers to participate in the survey, specified that the person(s) responsible for Clinical Unit/Department in the company, should complete the survey. The questionnaire, which was validated by two professionals not involved in the survey, contained 74 questions specifically designed to collect information related to four main areas of clinical development function: (1) the organizational structure and the activities conducted by the clinical functions of the company; (2) the trial design ability and the management of clinical trial documents; (3) the clinical trial management and monitoring activities; (4) the quality aspects of clinical activities.

The manufacturers voluntarily filled in the online survey, and results were analysed using SurveyMonkey®.^3^

No statistical hypothesis had been formulated when the study was designed, hence, only semi-quantitative observation of trends was done without statistical confirmation. Filled in questionnaires were received electronically in anonymous way and were analysed as such, in a qualitative manner.

## Results

From 41 manufacturers contacted, three manufacturers informed the DCVMN secretariat that they would not participate in the survey, as their companies do not have any such clinical activities. A total of 33 filled-in questionnaires were found in the database at the time of survey database lock. At database cleaning, it was found that 3 respondents were in duplicates, as identified by the same IP address. Duplicates were excluded by removing the questionnaire filled in at a later time. A total of 30 respondents (unique IP address) filled in the questionnaire, of which 25 responded to all questions. In conclusion a total of 33 from 41 DCVMN members provided feed back to the survey (80% response rate) of whom 3 had no clinical activities ongoing. The final analysis was performed on the data derived from 30 respondents. The analysis of trends to each question was performed using ratio analysis, using as denominator the number of responders to that specific question, and numerator was the number of positive answers to the same question. As overall numbers were small, a descriptive analysis is presented here.

### Organizational structure and the activities conducted by the clinical functions of the company

All respondents (n = 30) indicated to have a clinical department which included the wording “clinical”, “development” or “research”. The slight majority of respondents (17/30) reported to have more than 10 employees in the clinical department and almost all respondents have employed either one medically qualified doctor (MD) or one Doctor of Philosophy (PhD). About half of all the respondents with 10 or less than 10 employees (n = 13) have either one MD or PhD (Table 1).

**Table 1:** Proportion of MD/PhD presence as to size of Clinical Affairs Department/Unit.

Total number of employees in clinical affairs	Presence of at least one MD (%)	Presence of at least one PhD
1–10 employees (13 respondents)	7/13 (54%)	6/13 (46%)
>10–20 employees (8 respondents)	7/8 (88%)	7/8 (88%)
>20 employees (9 respondents)	7/9 (78%)	7/9 (78%)
Total respondents	30	30

DCVMN respondents categorized in three groups as to total number of employees in clinical department (column 1) as small, medium and large groups, and stratification by presence of at least one MD or PhD. MDs and PhDs appear to be present in around 80% of the companies with medium or large clinical teams, and only present in about 50% of the companies with smaller (1–10 employees) clinical teams.

**Fig. 1:**
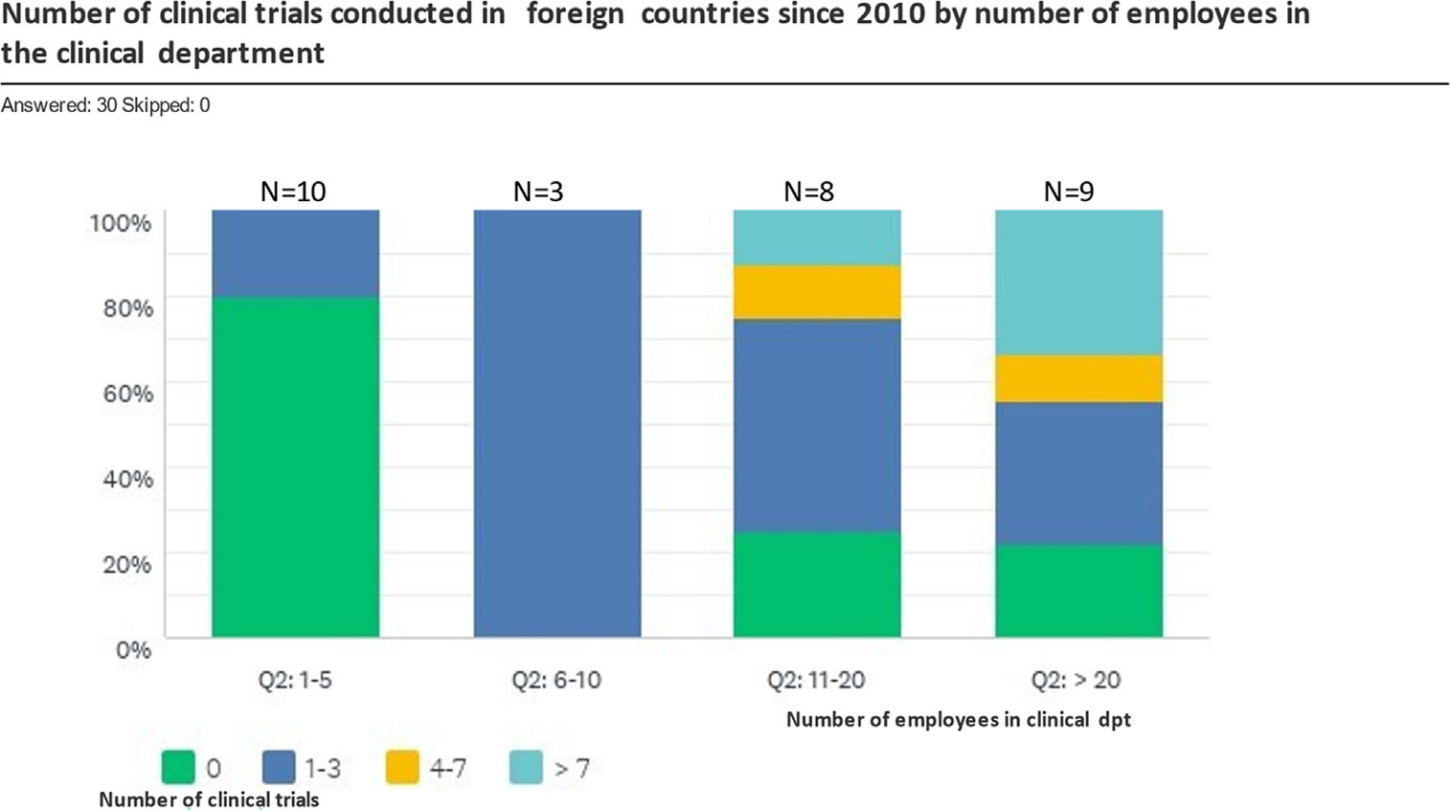
Number of clinical trials conducted in foreign countries since 2010, stratified by number of employees in the clinical department. Fig. 1. Each column relates to the number of employees in the clinical department/unit, or clinical department/unit size from left to right: first column 1–5 employees; second column 6–10 employees; third column 11–20 employees; forth column over 20 employees. Number of respondents within each category is indicated on the top of each column. (N = X). The column colours indicate the number of clinical trials conducted abroad as proportion of respondents within each group. 80% of small clinical departments/units did not conduct clinical trials abroad in the past ten years (green bar in first column) while 80% of clinical departments/units with over 20 employees did have clinical trials abroad, and 50% of those had conducted more than 4 trials abroad. (For interpretation of the references to colour in this figure legend, the reader is referred to the web version of this article.)

Almost all respondents had at least one or more vaccines licensed in the national market (29 respondents) or in foreign markets including vaccines with World Health Organization Prequalification (WHO PQ) status (27 respondents). The majority of respondents (24/30) had at the moment of the survey one or more vaccine in clinical development, and since 2019 over 70% of them has initiated at least one clinical trial. Overall, 60% or more respondents had at least 2 presently ongoing clinical trials. Observed values for the total number of employees and the number of ongoing clinical trials did not suggest an association between these two indicators, except for respondents with up to 5 employees in the clinical unit, most of whom did not have any clinical trial ongoing or started since 2019. A total of 40% (12/30) of respondents have not conducted any clinical trial in foreign countries since 2010, and this activity seems to depend on the demand for supply internationally, which will drive the need for the higher number of employees in the clinical department (Fig. 1).

Since 2015, 90% (27/30) of respondents has submitted at least one Common Technical Document (CTD) to NRAs, and 60% (18/30) submitted more than 3 CTDs. The number of submissions of CTDs since 2015 to obtain WHO PQ were lower than national submissions, with 62% (18/29) of respondents having submitted at least one CTD, and 28% more than 3 since 2015.

**Fig. 2:**
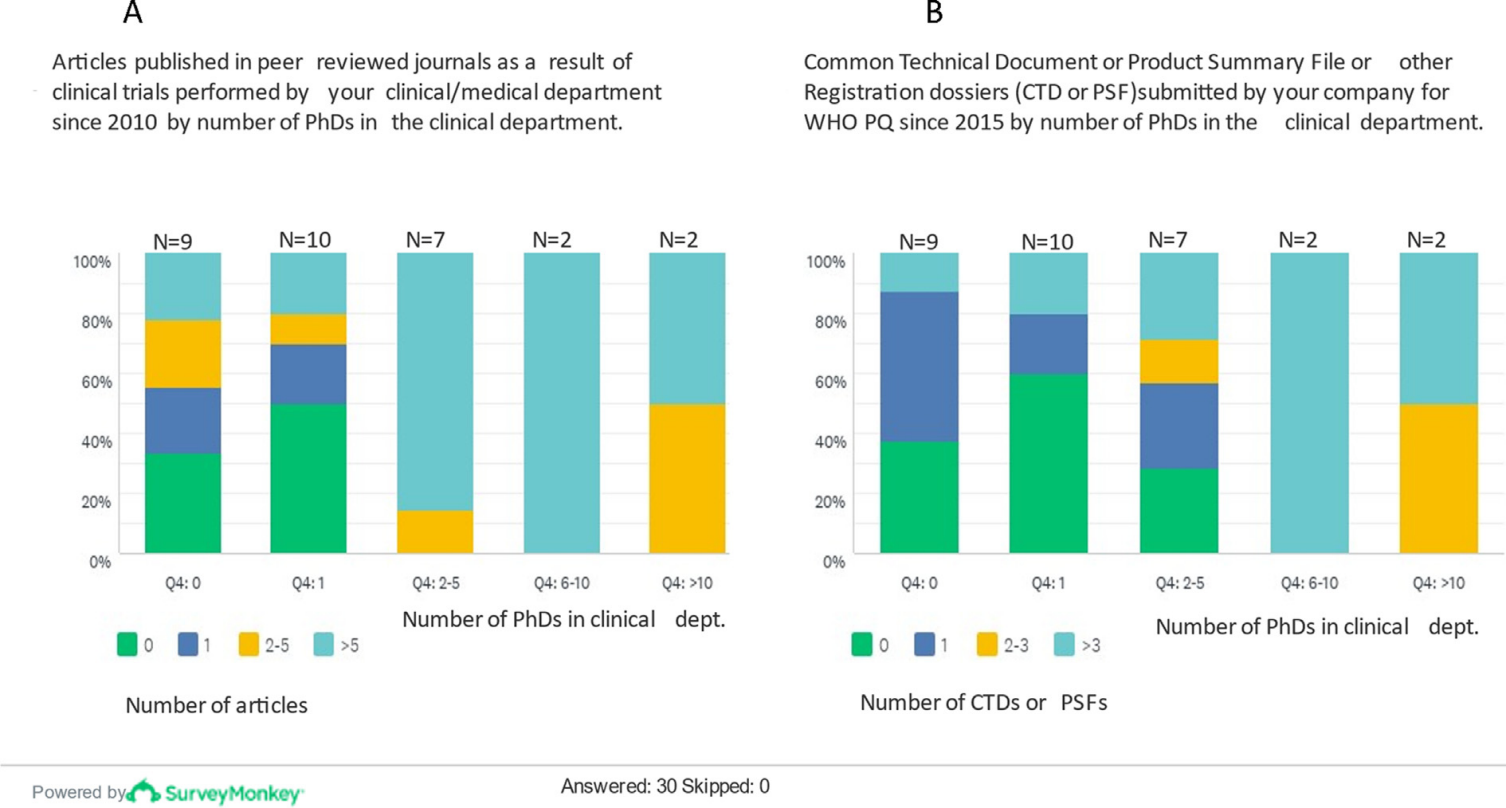
Scientific publications and WHO submissions as related to number of PhDs in the clinical department/unit. Fig. 2A. Number of articles published in peer-reviewed scientific journals in the last 10 years stratified by number of PhDs in the clinical department. The number of respondents (N) to each range of PhDs employed in the clinical department is indicated on the upper part of each column. The number of PhDs in the clinical department varied from 0 to over 10, and the higher the number of PhDs employed, the more articles were published in peer-reviewed scientific journals. Notably, 35–50% of clinical departments/units with none or only one PhD, did not publish any article in the last 10 years, as depicted by the green areas on the first and second columns, whereas 100% departments with 2 to 10 PhDs employed, published often more than 5 papers in the same period. Fig. 2B. Number of CTDs submitted to WHO in the last 5 years stratified by number of PhDs in the clinical department. The number of respondents (N) to each range of PhDs employed in the clinical department is indicated on the upper part of each column. The number of CTDs (or PSF) submitted to WHO prequalification appear higher in clinical departments/units that employ more than 2 PhDs, as depicted by the yellow and light blue areas of the three right columns on this chart. (For interpretation of the references to colour in this figure legend, the reader is referred to the web version of this article.)

Since 2010, 73% (22/30) of respondents had published at least one article in peer reviewed journals with data derived from clinical trials, and 43% (13/30) published more than 5 articles, though corporate publishing policy about clinical trials results was not assessed in this survey. This publishing activity, together with the number of CTDs submitted to WHO since 2015, seems to indicate a positive trend with the need for higher number of PhDs in the clinical department (Fig. 2). Nevertheless, it may well be that the proportion of MD/PhD is the same for small and larger teams, thus the number of publications may also indicate a larger clinical team.

### Trial design ability and management of clinical trial documents

**Fig. 3:**
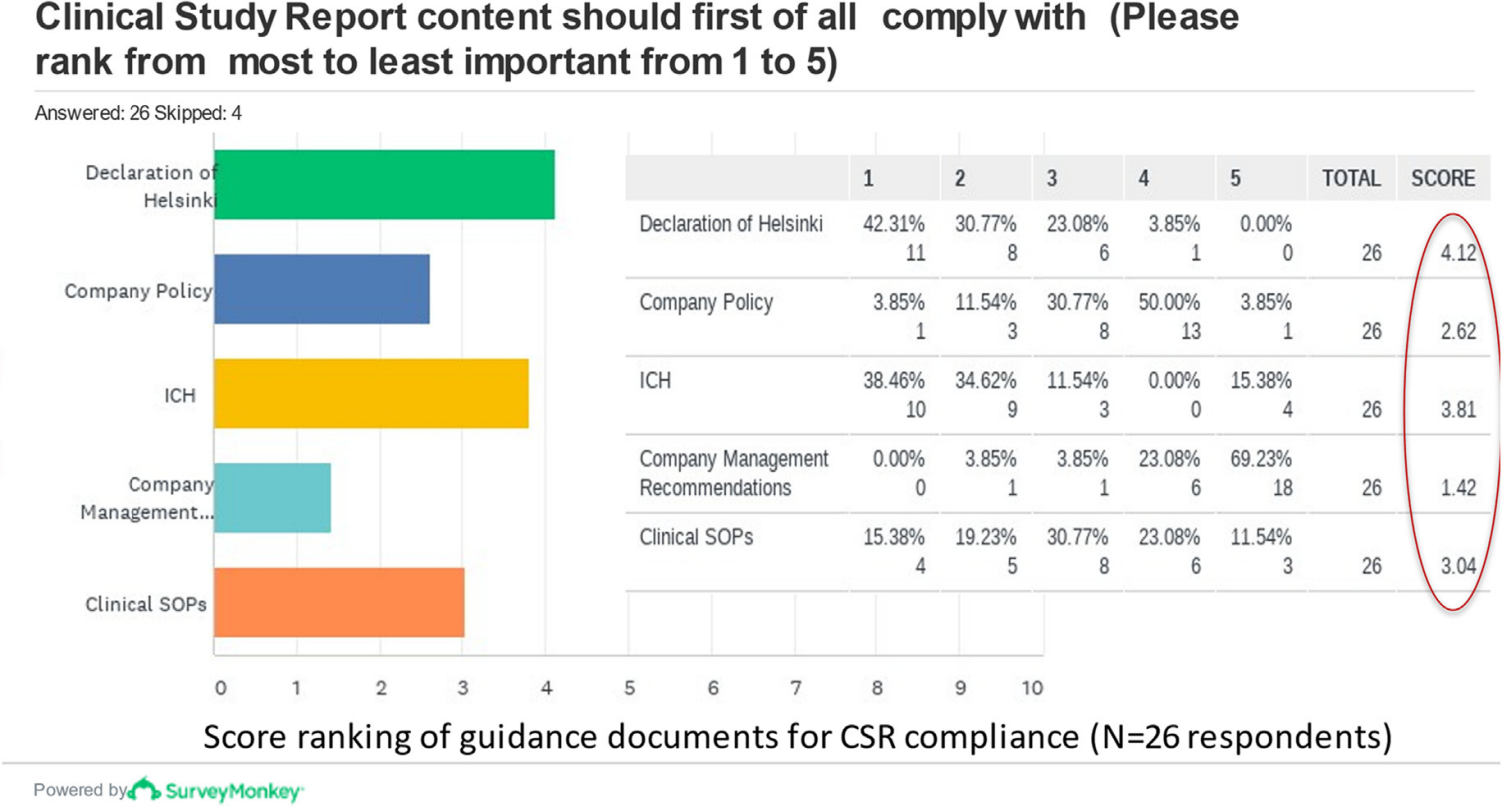
Guidelines/guide documents content CSR should comply with, as prioritized by respondents. Ranking in order of importance of guidelines/documents content that CSR should comply with, as to manufacturers’ responses. From 26 respondents to this survey question (4 kept this question), the Declaration of Helsinki was ranked first (42%), the ICH guidelines ranked second (39%), clinical SOPs ranked third (15%) and company policy ranked fourth (4%), with no importance assigned to corporate management. Items ranked first are given a higher value or “weight”. The score is a weighted calculation, computed for each answer option, as the sum of all the weighted values. The weighted values are determined by the number of columns, which is the number of options available.

All respondents to this questions’ section reported initiating clinical trials only after approval by Ethic Committees (ECs) (Fig. 3), and the majority (18/26) of respondents always write a clinical development plan for candidate vaccines to enter in clinical development. As it is not expected that studies are initiated prior to ethical approval, the level language skills to understanding of the English questionnaire may have influenced and limited the information, or lack of it, provided by some respondents. Some respondents did not answer this question, perhaps due to lack of knowledge or understanding, however nobody claimed to start any clinical study without approval. About 56% (14/25) of respondents set one primary objective in designing a clinical trial and 44% (9/25 respondents) set between 2 and 3 primary objectives.

The majority of respondents 85% (22/26) always collect records of Adverse Events (AEs) and Serious Adverse Events (SAEs) in clinical trials. However, the length for collecting solicited and unsolicited AEs, and SAEs are usually collected in a clinical setting “according to the vaccine”, reflecting a product specific approach.

A total of 85% (22/26) respondents stated that clinical trial participants or parents/legal guardians’ participants should sign the Informed Consent Form before the start of any study procedure or screening procedure and 88% (23/26 respondents) always register clinical trials in publicly available clinical trials platforms.

The majority of respondents (21/26) stated to amend study protocols on average at least once during the study period, and 35% (9/26) stated to write between 3 and 4 protocol amendments. All respondents (25/25) stated to have the Statistical Analysis Plan (SAP) finalized before the clinical trial database is completed and locked for statistical analysis, and 72% (18/25) have the Statistical Analysis Report (SAR) finalized before the process of writing the Clinical Study Report (CSR) is initiated. The CSR content is well described in ICH-E3 and it should be object of a dedicated clinical standard operating procedures (SOPs). When asked to rank in order of importance the guidelines/documents the CSR content should comply with the Declaration of Helsinki ranked first and ICH second. (Fig. 3).

### Clinical trial management and monitoring activities

The majority of respondents perform different clinical trials activities by always or sometime outsourcing to Clinical Research Organisations (CROs). A total of 88.5% (23/26) respondents always delegate the selection of clinical study sites to CROs, or sometimes delegate (16/26 or 62%). Clinical trial protocols are always written in house by 48%respondents (12/25), sometimes by 44% (11/25) and never by 8% (2/25). CSRs are written in house by the majority of respondents (19/25 or 76 %), and the Investigator Brochure (IB) is written in house by 96% (25/26). A minority of respondents (4/25 or 16%) always perform clinical monitoring activities in house, 32% (8/25) always outsources this activity and 52% (13/25) do it sometimes. Clinical data management and statistical activities are always outsourced by 50% (13/26) of respondents, and are sometimes outsourced by 42% (11/26). In the past five years, 46% (12/26) of respondents have employed between one and two CROs, 35% (9/26) between three and four, and 19% (5/26) more than four.

**Fig. 4:**
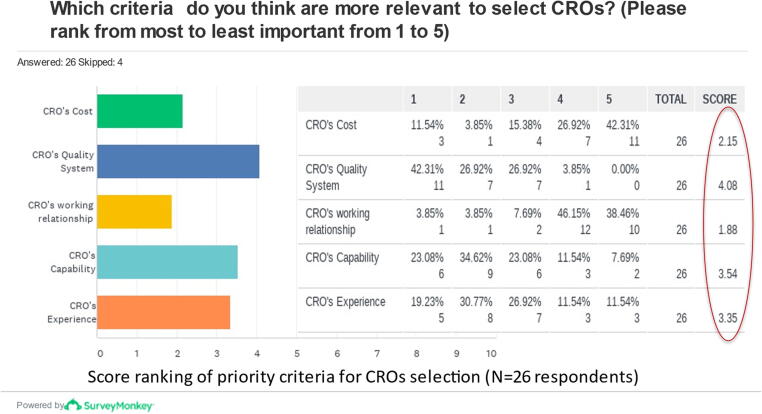
Criteria adopted to select a CRO in order of importance. Quality systems was ranked first (42%), followed by capability (23%), previous experience (19%), costs (12%) and relationship was last (4%) in order of importance of criteria to select a CRO, as ranked by 26 respondents to this specific question (4 kept). Items ranked first are given a higher value or “weight”. The score is a weighted calculation, computed for each answer option, as the sum of all the weighted values. The weighted values are determined by the number of columns, which is the number of options available.

46% (12/26) of respondents always performs audit to CROs, 42% (11/26) does it sometimes, and 12% (3/26) never does it. The criteria adopted by respondents to select CROs are reported in order of importance as depicted in Fig. 4. The highest-ranking criteria are the CRO’s quality system, the CROs capability and the CROs experience.

**Fig. 5:**
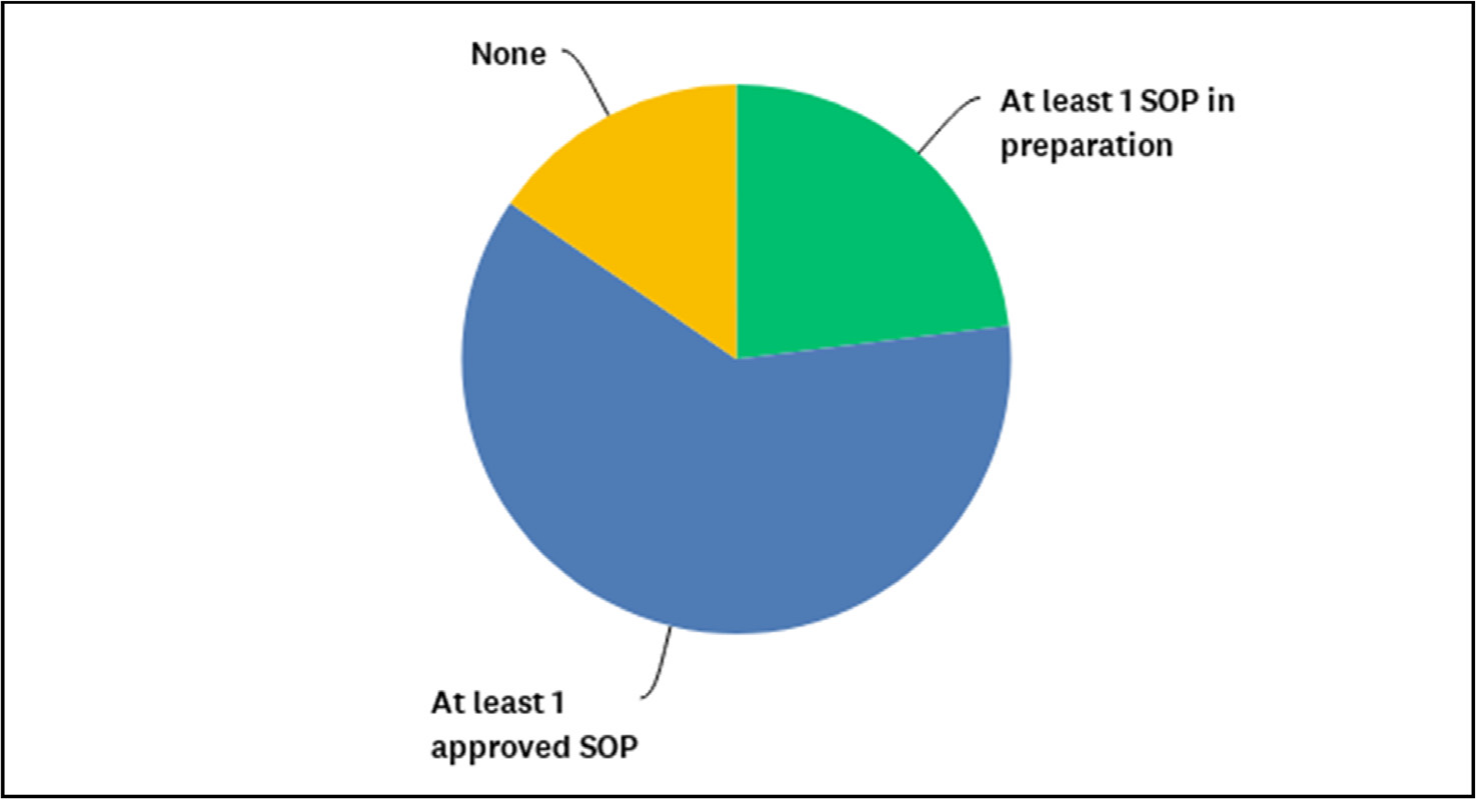
Availability of SOPs on how to select and manage CROs. Three (12%) out of 26 respondents had no SOP available on how to select CRO, at the time of the survey, while 5 (19%) had SOPs in preparation and 18 (69%) had selection SOPs readily available.

The great majority of respondents (81% or 21/26) are satisfied with the CROs they have engaged in the past 5 years. However, 58% (14/26) believe that CROs need to be supervised by the sponsor to be able to comply with ICH-GCP. The vast majority of respondents (88% or 22/26) stated to have one or more clinical SOP approved or in preparation on how to select and manage CROs (Fig. 5).

Laboratory assays to evaluate immunogenicity in human sera of candidate vaccines are always performed by an “in house” company laboratory by 35% (9/26) of respondents, while sometimes this activity is outsourced by 58% (15/26) of respondents and always outsourced by 8% (2/26). Qualification visits to external laboratories are always performed by 39% (10/26) of respondents, sometimes by 58% (15/26) and only one respondent never did it. The most important criteria used to select an external laboratory include: first, to be a WHO reference laboratory; second, to have an established collaboration and third, that the laboratory is known for the scientific publications.

### Quality aspects of the clinical department

The vast majority of respondents (92% or 23/25) has an approved organizational chart for the clinical department, as well as a job description for each employee, and 80% (20/23) has up-to-date CVs and training records for each employee. The majority of respondents (68% or 17/25) had the master clinical SOP and none of the responders had SOPs for all aspects concerning clinical development.

Review of SOPs was performed by 36% (9/25) of respondents “when changes are required in one or more SOPs”, 20% (5/25) reviewed SOPs every three years, 36% (9/25) every two years, and 8% (2/25) did it every year. About 52% (13/25) of respondents had a clinical SOP on how to audit a CRO, and 24% (6/25) had clinical trial sites audited by an independent auditor in the past 5 years. The majority of respondents (88% or 22/25) had one or more clinical trial sites inspected by the NRA. However, 60% (15/25) had a clinical SOP on “how to handle clinical trial audits and inspections”; the others either did not have any (20% or 5/25) or had one in preparation (20% or 5/25).

**Fig. 6:**
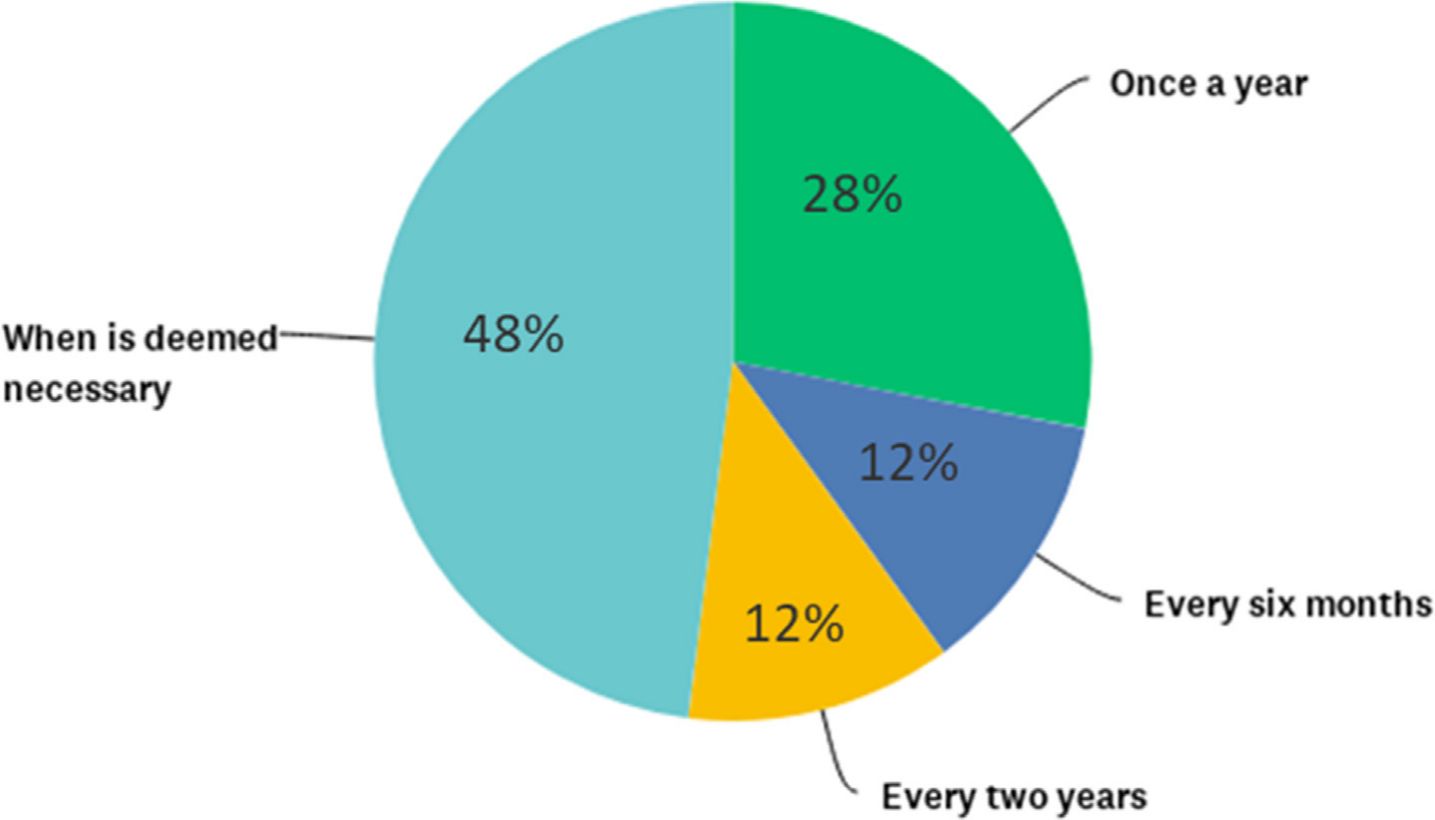

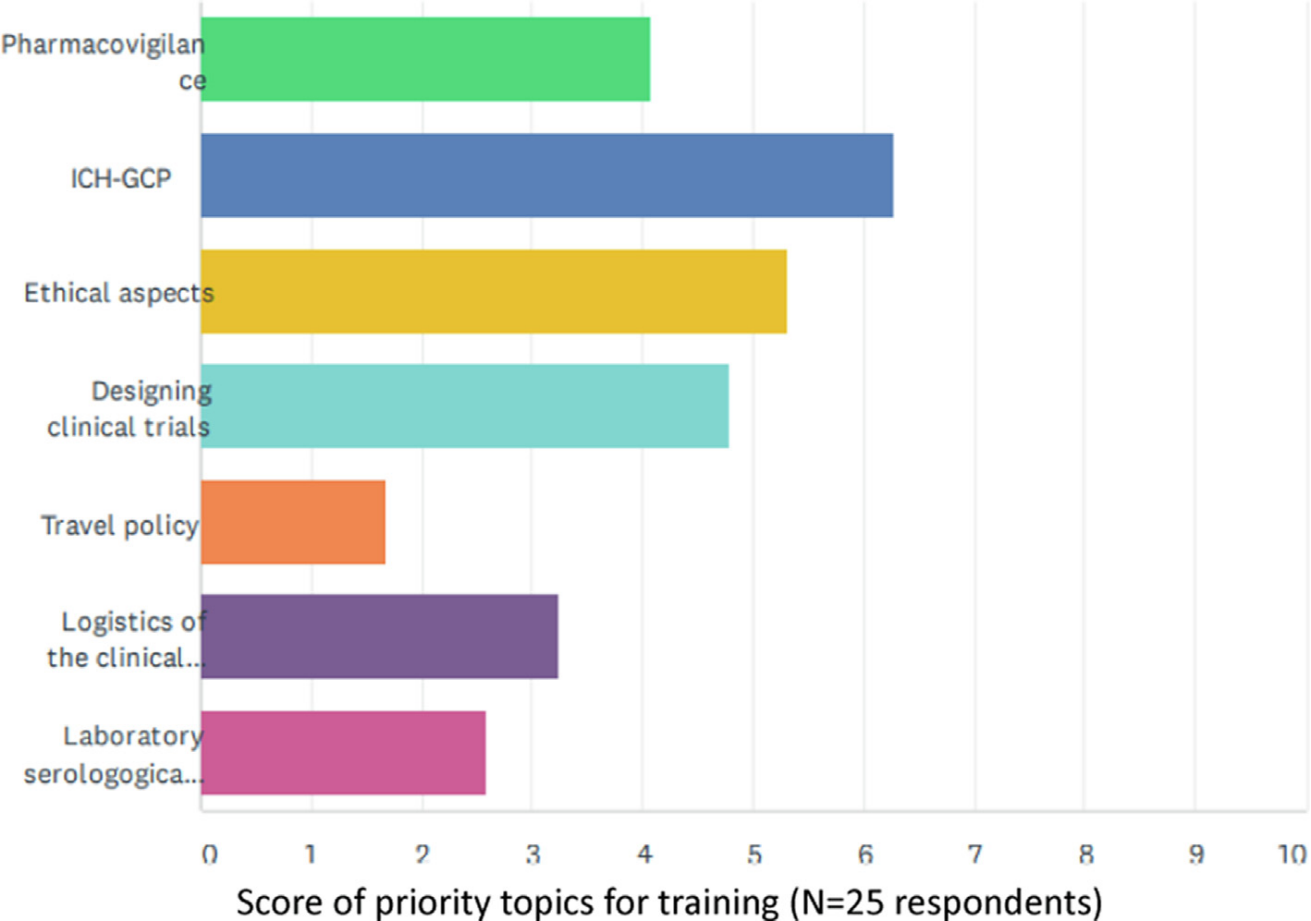
Frequency and perception of priority topics to specific training delivered to clinical department employees. Fig. 6A. Approximately half of the respondents consider to provide training on GCP and Pharmacovigilance when deemed necessary. (N = 25) Some skipped this question (N = 5). Fig. 6B. Prioritization of clinical training topics to be delivered to clinical department employees in order of importance, according to 25 respondents. Highest priority was assigned to ICH-GCP, followed by ethics, design of clinical trials, and pharmacovigilance. Lower priority was assigned to logistics, laboratory assays and lowest to travel policy, in the context of this question as related to clinical activities.

Regular training to clinical department employees, concerning specific aspects of ICH-GCP, or PV or other scientific and operational topics, was delivered by 48% of respondents (12/25) “when deemed necessary”, 28% (7/25) delivered the training once a year, 12% (3/25) delivered training every two years, and every six months (Fig. 6A). Fig. 6B shows the topics of training to be considered priority for clinical personnel involved in conducting clinical trials, as to 25 respondents.

The highest importance is attached to training on Good Clinical Practices (GCP) aspects, together with training on ethical aspects, that are also part of ICH-GCP. The training on aspects of clinical trial design and on pharmacovigilance ranked third and fourth as importance, as considered in this survey.

## Discussion

The urgent need to stop the spread of infectious diseases, to control epidemic and pandemic events has transformed vaccine development timelines, reducing the timelines of clinical phases of development in order to save lives [13]. The clinical development of vaccines against infectious diseases requires expertise in many areas, including preclinical stages, clinical study design, manufacturing processes, laboratory quality control assays, disease surveillance [14], regulatory pathways, supply-chain, safety monitoring and public immunization programmes [15].

This survey focused on assessing clinical development practices, rather than policies, as indicator of preparedness among vaccine manufacturers in emerging countries [16]. The majority of respondents have reported accurate practices in the ethical and methodological aspects of clinical trial design, as well as in the management of clinical trial documents. However, the language skills to understanding of the English questionnaire may have influenced and limited the information, or lack of it, provided by some respondents. Noteworthy, it has been observed that the number of clinical trials conducted in foreign countries since 2010 (see Fig. 1) seems to be higher when more than 10 employees are working in manufacturers’ clinical departments. Conducting clinical trials in foreign countries may require additional resources to overcoming many barriers such as language barriers, difficulties in establishing relations with local partners, complexity of clinical trial documentation, logistics limitations, and knowledge of local regulations and requirements. The financial resources required in each clinical trial of vaccines, as well as the total number of clinical studies, was not specifically assessed in this survey, because it is a complex question requiring a specific survey on budgeting/resources addressed to financial managers/departments.

In this survey the number of PhDs employed and the size of the clinical department suggested a trend related to some indicators of clinical activities, such as the number of published articles in peer-reviewed journals since 2010, the number of CTDs submitted to WHO for PQ in the last five-years’ period^4^, and the approach to methodological aspects of the clinical trial design. Publishing aggregated clinical studies data is an indicator of transparency and serves as scientific information source for health authorities, health professionals, scientific community and the public in general about the vaccines supplied globally. In addition to number of highly qualified personnel, there could be other factors influencing the volume of scientific publications, such as size of the company, number of years the company has been active, number of licensed vaccines, and other aspects that have not been captured in this survey, due to the need to preserve anonymity of respondents. The boundary between private sector research and traditional academic research is blurred, to distinguish research with generalisable benefits for society from that which serves private interests. Corporate secrecy can also represent a barrier to publish scientific research [17]. Nevertheless, these findings highlight the importance for vaccine manufacturing companies of engaging qualified staff such as PhDs or MDs in all clinical related activities. While the majority of respondents retain suitable practices in the ethical and methodological aspects of clinical trial design, as well as in the management of clinical trial documents, some areas of improvements for international supply were identified including the definition and collection of AEs and SAEs during clinical trials, in the informed consent form (ICF) process, on the obligation to register clinical trials in publicly accessible registries and in the CSR structure.

The great majority of respondents outsourced one or more clinical trial management activities to CROs, such as selection of study sites, monitoring and data management, while clinical trial protocols, investigator brochure and clinical reports are mostly performed in house. On the other hand, clinical data management and statistical analyses are clinical activities outsourced by around half of the respondents. In this context, some areas for improvement identified by this survey include the standard process of selecting and managing CROs, as well as verifying through audits whether CROs have a quality system in place. While the majority of respondents is satisfied with the performances of the CROs employed, some believe that Sponsor’s supervision is required for CROs to comply with ICH-GCP.

Although the majority of respondents retains a clinical quality system in place with clinical SOPs, none has SOPs for all the typical activities of clinical development. Specific training to the clinical staff is delivered by a large proportion of responders “when deemed necessary”, while ICH-GCP and PV training should be given at least once a year [18]. GCP and Pharmacovigilance regular training is a requirement for all professionals involved in clinical trials. In some countries, the non-compliance with ICH GCP E6(R2) regulations results in inspection findings, cost overruns, data rejection, clinical study delays and potential delays in access to live-saving vaccines. Research also shows that trainees only retain 20% to 30% of verbal information whereas approaches including simulation / experiential learning increase information retention to 70% up to 90% [19] thus the need for more practical training. Respondents themselves ranked in order of importance which topics the training should include as follows: ICH-GCP, Ethics, Study Design methodology and PV. Therefore, it is inferred that fostering a dynamic and large R&D portfolio of candidate vaccines, maintaining staff hands-on training and proficiency in new technology and laboratory assays’ implementation, pre-clinical studies, as well as phase 1, formulation, dose-finding and tolerability studies, will enable staff preparedness to rapidly tackle phase 2 and 3 studies, when needed.

## Conclusion

The trend to accelerate vaccine clinical development was reinforced with the global COVID-19 pandemic in 2020 [20] and the first COVID-19 vaccines became available to the public just nine months after the declaration of the pandemic [21], [22]. The Ebola outbreak in West Africa, in the spring of 2014, already showed the global health community that it was possible to compress clinical development timelines for vaccines to less than 12 months [23]. Particularly, several vaccine manufacturers from emerging countries were able to bring COVID-19 vaccines to markets within a year [24]. These recent global experiences of fast-tracking clinical development could now be adopted for other clinical studies of new vaccines in emerging countries. DCVMN as a network is also seeking to encourage existing harmonization efforts in regulatory practices that accelerate access to new vaccines [25], [26], [27], through fostering dialogue with NRAs and information exchange among manufacturers based in different countries.

Clinical development of candidate vaccine is a complex process that requires specific expertise in different fields and the construction of solid operational and quality systems. The results of this survey among some vaccine manufacturers in emerging countries suggest that areas to further strengthen the clinical development and medical research preparedness include the following:

• consider increasing engagement of MD and PhD qualified staff in the clinical department, in several clinical activities (i.e. scientific articles, CTD submissions for WHO PQ);
• consider that network members voluntarily adhere to publishing trials results, i.e. to publish at least one paper for each clinical trial;
• improve the systems to collect and report AEs and SAEs during clinical trials;
• improve ICF process;
• improve CSR compliance;
• improve the system on how CROs are selected, managed and supervised, with specific SOPs,
• improve the clinical quality system in place by completion of all clinical SOPs, and frequent independent clinical audits,
• deliver periodic training as identified by the respondents themselves in order of importance: ICH-GCP (at least once a year), Ethics, Study Design methodology and pharmacovigilance.

Implementing the above recommendations will require higher stakeholders’ engagements from oversight bodies and investments and resources from manufacturers to expand the clinical basis in vaccine R&D, to support achieving a high level of preparedness in emerging countries for accelerated development of new vaccines against future epidemics and pandemics. The Network, as a professional association, is committed to facilitate clinical training to ensure that manufacturing staff members are informed, adhere and conform to quality clinical development activities.

## Disclaimer

The authors alone are responsible for the views expressed in this article, which do not necessarily represent the views, decisions or policies of any institutions with which the authors are associated.

## Declaration of Competing Interest

The authors declare that they have no known competing financial interests or personal relationships that could have appeared to influence the work reported in this paper.

## Data Availability

Data will be made available on request.

## Footnotes

1 https://cioms.ch/publications/product-category/recently-published/.

2 https://www.dcvmn.org/Members-126.

3 https://www.surveymonkey.com.

4 https://extranet.who.int/pqweb/vaccines/prequalified-vaccines.
